# Small and Medium-Sized Aneurysm Outcomes Following Intracranial Aneurysm Treatment Using the Pipeline Embolization Device: A Subgroup Analysis of the PLUS Registry

**DOI:** 10.3389/fneur.2022.881353

**Published:** 2022-05-31

**Authors:** Hongyun Zhang, Li Li, Hongqi Zhang, Jianmin Liu, Donglei Song, Yuanli Zhao, Sheng Guan, Aisha Maimaitili, Yunyan Wang, Wenfeng Feng, Yang Wang, Jieqing Wan, Guohua Mao, Huaizhang Shi, Bin Luo, Qiuji Shao, Kaitao Chang, Qianqian Zhang, Yingkun He, Peng Zhang, Xinjian Yang, Tian xiao Li

**Affiliations:** ^1^Cerebrovascular Department of Interventional Center, Henan Provincial People's Hospital, Zhengzhou University People's Hospital, Henan University People's Hospital, Zhengzhou, China; ^2^Neurosurgery Department of Stroke Center, Henan Provincial People's Hospital, Zhengzhou University People's Hospital, Henan University People's Hospital, Zhengzhou, China; ^3^Department of Neurosurgery, International Neuroscience Institute (China-INI), Xuanwu Hospital, Capital Medical University, Beijing, China; ^4^Department of Interventional Neuroradiology, Xuanwu Hospital, Capital Medical University, Beijing, China; ^5^Changhai Hospital Affiliated to Naval Medical University, Shanghai, China; ^6^Shanghai Donglei Brain Hospital, Shanghai, China; ^7^Peking University International Hospital, Beijing, China; ^8^First Affiliated Hospital of Zhengzhou University, Zhengzhou, China; ^9^First Affiliated Hospital of Xinjiang Medical University, Ürümqi, China; ^10^Qilu Hospital of Shandong University, Jinan, China; ^11^Nanfang Hospital, Southern Medical University, Guangzhou, China; ^12^First Affiliated Hospital of Nanchang University, Nanchang, China; ^13^School of Medicine, Renji Hospital, Shanghai Jiao Tong University, Shanghai, China; ^14^Second Affiliated Hospital of Nanchang University, Nanchang, China; ^15^First Affiliated Hospital of Harbin Medical University, Harbin, China; ^16^Beijing Neurosurgical Institute, Beijing Tiantan Hospital, Capital Medical University, Beijing, China

**Keywords:** endovascular, pipeline embolization device, small and medium-sized, intracranial aneurysm, multi-center study, complications, outcome

## Abstract

**Objective:**

The purpose of this work was to summarize the real-world safety and efficacy of Pipeline Embolization Device (PED) therapy for small and medium-sized intracranial aneurysms in China.

**Methods:**

Patients from the PED in China post-market multi-center registry study (PLUS) with aneurysms smaller than 12 mm were selected. Radiographic outcomes were assessed using digital subtraction angiography. Clinical outcomes included functional outcomes (modified Rankin Scale, MRS) in the early postoperative period ( ≤ 30 days) and early postoperative complications associated with PED therapy.

**Results:**

A total of 652 patients with a combined 754 aneurysms were included in this study (mean age of 53.9 ± 10.3 years, 68.7% women). Mean aneurysm diameter was 6.78 ± 2.67 mm. Of the 687 stents deployed, 99.7% (685/689) were successfully deployed. In this study, 64.7% (488/754) of aneurysms were treated with only the PED, whereas 35.3% (266/754) were subjected to PED-assisted therapy. Radiographic outcome at the last follow-up (median time: seven months) was available for 64.3% (485/754) of the aneurysms. 82.5% (400/485) of aneurysms demonstrated complete occlusion (Raymond Roy Grade I). 81.4% (395/485) of aneurysms were found to meet the study's primary effectiveness outcome. At the early postoperative period, the mRS score was determined to be 0–2 vs. 3-6 in 98.2% (640/652) vs. 1.8% (12/652) of the cases, respectively. The combined major morbidity and mortality rate was 3.2% (21/652).

**Conclusion:**

In the largest study of PED therapy for small and medium-sized intracranial aneurysms to date, pipeline-assisted coil embolization was chosen more often than multiple stent implantation for aneurysm treatment, demonstrating good results, high surgical success rates, high occlusion rates, and low morbidity and mortality.

**Clinical Trial Registration:**

www.ClinicalTrials.gov, identifier: NCT03831672.

## Introduction

Flow-diverting stents provide a new strategy for the treatment of aneurysms. This type of stent is placed in the parent artery at the level of the aneurysm neck to divert blood flow away from aneurysm, thus contributing to the gradual formation of aneurysmal thrombosis. Furthermore, flow-diverting stents provide scaffolding for the growth of endothelial cells in the aneurysm neck (1, 2). In 2011, the US Food and Drug Administration (FDA) approved the use of the flow-diverting Pipeline Embolization Device (PED) for treatment of large or giant wide-neck intracranial aneurysms (IAs) involving the petrous to the superior hypophyseal segments (3). However, large and giant aneurysms account for only a small fraction of all IAs, whereas small and medium-sized unruptured IAs account for ~80% (4). Moreover, the majority of ruptured IAs are smaller than 10 mm, and the annual rupture rate of small aneurysms is 0.5%-2% (4, 5). In the PREMIER study, small and medium-sized ( ≤ 12 mm) wide-neck aneurysms had a high rate of complete occlusion and low rate of associated permanent neurological complications (6). However, the results of PED therapy among large samples of patients with small and medium-sized intracranial aneurysms have rarely been reported. PLUS was a multi-center, panoramic, consecutive, real-world cohort registry study designed to evaluate the safety and effectiveness of PED treatment in the embolization of IAs among the Chinese population (7). Compared with previous PED studies (see Supplementary Table 1), PLUS study provided us with an opportunity to assess the safety and effectiveness of the treatment after flow diversion for small and medium-sized intracranial aneurysms. In this study, we reported the safety and effectiveness of PED treatment after flow diversion for IAs from the PLUS study that were smaller than 12 mm.

## Methods

### Study Participants

Details of the inclusion and exclusion criteria were described in the original PLUS study (7). In the PLUS study, patients with intracranial aneurysms who were treated with the PED at 14 centers in China between November 2014 and October 2019 were enrolled. A total of 1,171 patients with a combined 1,322 intracranial aneurysms were included in the PLUS study. In this work, we retrospectively evaluated patients with aneurysms smaller than 12 mm in the PLUS study. A total of 652 patients with a combined 754 aneurysms were included in this study. In this study, 652 procedures were performed to treat the 754 aneurysms studied. Both ruptured and unruptured aneurysms are treated.

### Baseline Assessments

Baseline neurological assessment was performed prior to surgery using the Modified Rankin Scale (mRS) and the National Institutes of Health Stroke Scale (NIHSS). Preoperative CT angiography, MR angiography, or Digital Substraction Angiography(DSA) was performed to complete the baseline imaging assessment.

### Dual Antiplatelet Therapy

Patients received an antiplatelet regimen that included a combination of aspirin (100–300 mg/day) and clopidogrel (75 mg/day) 3–5 days before surgery. Additionally, some patients underwent thromboelastogram (TEG) testing. Patients identified as nonresponsive to clopidogrel were given aspirin (100 mg/day) and ticagrelor (90 mg twice daily). Among each of the centers, the duration of dual-antiplatelet therapy after surgery varied from 3–6 months. After this, Aspirin was used indefinitely.

### Procedure Details

The device used in embolization procedures included the PED Classic or PED Flex. The PED was delivered and deployed via a Marksman microcatheter (Medtronic; Irvine, California). The use of coils was determined by the treating physician. Intravenous heparin was administered at 50–100U/kg to achieve an activated clotting time >250s.Aneurysm occlusion was determined using digital subtraction angiography. The degree of aneurysm occlusion was assessed using the Roy Raymond Scale (8).

### Study Outcomes

The primary effectiveness outcome was complete occlusion of the target IA (Raymond Scale I) at the last radiographic follow-up without significant (≤ 50%) stenosis of the parent artery. The primary safety outcome included incidence of major stroke (ischemic or hemorrhagic) or neurologic death within 30 days of surgery. The secondary safety results included functional outcomes (modified Rankin Scale, MRS) in the early postoperative period (≤ 30 days).

All early postoperative complications were recorded, including major ischemic stroke (defined as an increase in the National Institutes of Health Stroke Scale [NIHSS]. score >4 lasting more than 7 days), minor neurological stroke (defined as a change in the NIHSS score of ≤ 4 lasting fewer than 7 days with corroborative imaging), transient ischemic attack (TIA, defined as a transient neurological deficit without corroborative imaging), delayed aneurysm rupture (DAR, rupture that occurs after surgery for the treated aneurysm), intracerebral hemorrhage (ICH), subarachnoid hemorrhage (SAH), stent migration, parent artery stenosis, and mortality.

Additional data collected included device deployment success rate and number of PEDs utilized. Dense packing was defined as complete occlusion, and loose packing was defined as incomplete occlusion.

### Statistical Analysis

Continuous variables, including demographics, aneurysm characteristics, and procedural characteristics, were represented as means and ranges. Categorical data were presented as numbers and percentages. Statistical analysis was performed using SPSS Version 25 (IBM Corporation; Armonk, NY, USA).

## Results

### Sample Demographics

Demographic and baseline characteristics are presented in Table 1. A total of 652 patients with a combined 754 aneurysms were included in this study. The mean age of the patients was 53.9 ± 10.3 years, and 68.7% (448/652) were female. The baseline mRS score was 0–2 vs. 3–6 in 99.1% (646/652) vs. 0.9% (6/652) of cases, respectively. Comorbidities included hypertension (42.9%, 280/652), diabetes (6.1%, 40/652), hyperlipidemia (4.4%, 29/652), coronary artery disease (6.0%, 39/652), cerebral atherosclerosis (16.4%, 107/652), alcohol abuse (10.9%, 71/652), and smoking (19%, 124/652).

**Table 1:** Baseline demographic characteristics.

**Demographics**	**Frequency (*N* = 652)**
Age (years) (mean ± SD)	53.9 ± 10.3
Female [*n* (%)]	448 (68.7)
Baseline NIHSS [mean ± SD (range)]	0.3 ± 1.0 (0–12)
Baseline mRS [*n* (%)]
0–2	646 (99.1)
3–5	6 (0.9)
Medical history [*n* (%)]
Hypertension	280 (42.9)
Hyperlipidemia	29 (4.4)
Diabetes mellitus	40 (6.1)
Coronary artery disease	39 (6.0)
Cerebral atherosclerosis	107 (16.4)
Alcohol abuse	71 (10.9)
Smoking history [*n* (%)]
Never smoked, or has not smoked within 10 years	480 (73.6)
Not a current smoker, but has smoked within 10 years	48 (7.4)
Current smoker, <1 pack/day	117 (17.9)
Current smoker, ≥1 pack/day	7 (1.1)

*NIHSS, National Institute of Health Stroke Scale; mRS, modified Rankin Scale; SD, standard deviation*.

### Aneurysm Characteristics

Aneurysm characteristics are presented in Table 2. The median aneurysm size was found to be 6.28 mm (mean 6.78 ± 2.67 mm), and the median neck size was 4 mm (mean 4.41 ± 1.98 mm). There were 83% (626/754) saccular, 3.6% (27/754) fusiform, 9.8% (74/754) dissecting, and 3.6% (27/754) blister aneurysms. Additionally, 3.8% (29/754) were ruptured aneurysms. A total of 629 (83.4%) aneurysms were located along the ICA. Most of the aneurysms were located at the C6 ophthalmic segment (47.7%, 360/629). 4.3% (32/754) of the aneurysms were located in the distal anterior circulation anteries (including the anterior and middle cerebral arteries), and 12.3% (93/754) were in the posterior circulation arteries.

**Table 2:** Aneurysm characteristics.

**Characteristics**	**Frequency (*N* = 754)**
Aneurysm size (mm)	6.78 ± 2.67
Neck width (mm)	4.41 ± 1.98
Dome to neck ratio	1.66 ± 0.69
Rupture aneurysm [*n* (%)]	29 (3.8)
**Aneurysm morphology [*****n*** **(%)]**	
Saccular	626 (83)
Fusiform	27 (4.4)
Dissecting	74 (9.8)
Blister	27 (3.6)
**Aneurysm location [*****n*** **(%)]**	
**Anterior circulation**	
Anterior circulation proximal	
ICA–clinoid segment (C4)	74 (9.8)
ICA–cavernous segment (C5)	112 (14.9)
ICA–ophthalmic segment (C6)	360 (47.7)
ICA–PCom segment (C7)	83 (11)
Anterior circulation distal	32 (4.3)
ACA	9 (1.2)
MCA	23 (3.1)
**Posterior circulation**	93 (12.3)
Vertebral artery	79 (10.5)
Basilar	10 (1.3)
Posterior cerebral artery	3 (0.4)
PICA	1 (0.1)

*ICA, internal carotid artery; ACA, anterior cerebral artery; MCA, middle cerebral artery; PICA, posterior inferior cerebellar artery*.

### Procedure

Procedural details are presented in Table 3. Of the 687 stents deployed, 99.7% were successfully deployed, of which 3.8% (26/687) stents were successfully deployed after technical adjustments. Complete wall attachment and complete neck coverage rates were 96.2% (725/754) and 99.7% (752/754), respectively. The mean number of devices used per patient was 1.01 ± 0.12 (range: 1–2). Multiple aneurysms were treated with one PED in 18.8% (142/687) of the patients. The PED Classic was used in 40.5% (278/687) while the PED Flex was used in 59.5% (409/687) of the patients. Of all aneurysms, 64.7% (488/754) were treated with the PED alone, whereas 35.3% (266/754) were treated with PED-assisted coil embolization. Among the aneurysms treated with the PED and coils, dense packing and loose packing accounted for 21.8% (58/266) and 78.2% (208/266), respectively.

**Table 3:** Procedural details.

**Characteristic**	***N* = 754**
Device deployment to the target site* **[*****n*** **(%)]**	
Successful	659 (95.9)
Successful after stent adjustment	26 (3.8)
Unsuccessful	2 (0.3)
Multiple aneurysms treated with one PED [*n* (%)]	142 (18.8)
Aneurysms with multiple PED [*n* (%)]	12 (1.6)
No of pipeline devices per patient [mean ± SD (range)]	1.01 ± 0.12 (1–2)
**Pipeline devices used [*****n*** **(%)]**	
Pipeline embolization device Classic	278 (40.5)
Pipeline embolization device Flex	409 (59.5)
Balloon used [*n* (%)]	8 (1.1)
PED alone [*n* (%)]	488 (64.7)
PED plus coil embolization [*n* (%)]	266 (35.3)
Loose packing	208 (78.2)
Dense packing	58 (21.8)
Complete wall apposition [*n* (%)]	725 (96.2)
Entire neck covered [*n* (%)]	752 (99.7)

*PED, Pipeline Embolization Device; SD, standard deviation*.

^*^The total number of PEDs used was 687.

### Primary Effectiveness Outcome

Angiographic outcomes are presented in Table 4. The mean imaging follow-up time was 8.26 ± 5.91 months (median time: 7 months; range: 3–36 months). Radiographic outcome at the last follow-up (median time: 7 months; range: 3–36 months) was available for 64.3% (485/754) of the aneurysms. At the last imaging follow-up, the primary effectiveness outcome was met in 81.4% (395/485) of the cases. Futhermore, 82.5% (400/485) of the aneurysms demonstrated complete occlusion (Raymond–Roy Grade I), and 1.4% (7/485) of parent arteries exhibited stenosis >50%.

**Table 4:** Angiographic outcomes.

**Characteristic**	**Frequency (*N* = 485 aneurysms)**
**Embolism result [*****n*** **(%)]**	
Complete occlusion	400 (82.5)
Residual neck	17 (3.5)
Residual aneurysm	68 (14)
**Parent artery status [*****n*** **(%)]**	
Patency	447 (92.2)
Stenosis ≤ 50%	31 (6.4)
Stenosis > 50%	7 (1.4)

### Safety Outcomes

The primary safety outcome included incidence of major stroke or neurologic death in the early postoperative period. The combined major morbidity and mortality rate was 3.2% (21/652). Among all patients, early postoperative major ischemic stroke occurred in 1.8% (12/652), major hemorrhagic stroke in 1.2% (8/652), and neurogenic death in 0.8% (5/652). The secondary safety outcomes included functional outcomes (modified Rankin Scale) in the early postoperative period. The mRS score during this period was 0–2 vs. 3–6 in 98.2% (640/652) vs. 1.8% (12/652) of cases, respectively.

### Complications

Table 5 presents the early postoperative complications. During the early postoperative period, 1.8% (12/652) of patients experienced major ischemic stroke and 4.0% (26/652) of patients suffered from minor stroke and/or TIA. Early postoperative hemorrhagic complications occurred in 2.0% (13/652) of patients, including SAH in 0.6% (4/652) and ICH in 1.4% (9/652). DAR was observed in 0.6% (4/652) of patients and stent migration occurred in 0.3% (2/652) of cases. The mortality rate was 0.8% (5/652). Overall, poor functional outcomes were observed in 1.8% (12/652) of the patients during the early postoperative period.

**Table 5:** Complications during the early postoperative period.

**Variable**	**Frequency (*N* = 652 patients)**
Major ischemic stroke	12 (1.8%)
TIA/minor stroke	26 (4.0%)
DAR	4 (0.6%)
ICH	9 (1.4%)
SAH	4 (0.6%)
Stent migration	2 (0.3%)
Mortality	5 (0.8%)
Poor functional outcome (mRS score: 3–6)	12 (1.8%)

*TIA, Transient ischemic attack; DAR, Delayed aneurysm rupture; ICH, intracerebral hemorrhage; SAH, Subarachnoid hemorrhage; mRS, modified Rankin Scale*.

## Discussion

In this study, patients from the PLUS study with aneurysms smaller than 12 mm were selected for analysis. A total of 652 patients harboring a combined 754 aneurysms (≤ 12 mm) were treated with PEDs at 14 centers in China between November 2014 and October 2019. This represents the largest study on PED treatment for small and medium-sized intracranial aneurysms to date.

In the PREMIER study (6), 81.9% of the patients (at 1 year) were Raymond–Roy Grade I, and 76.8% of patients were Raymond–Roy Grade I without major parent vessel stenosis (≤ 50%). In our study, the complete occlusion rate was relatively high compared to that of the PREMIER study, indicating that PEDs were used to treat small and medium-sized intracranial aneurysms with a high complete occlusion rate. At the last imaging follow-up (median time: seven months; range: 3–36 months), the primary effectiveness outcome was met in 81.4% (395/485) of the aneurysms, where 82.5% (400/485) demonstrated complete occlusion (Raymond–Roy grade 1). In a recent multicenter report of PED therapy for small aneurysms (≤ 7 mm), the rate of complete occlusion was 87% at the last imaging follow-up (median time: 6.5 months) (9). The above studies indicate that flow-diverting device have a high occlusion rate in the treatment of small and medium-sized aneurysms.

In line with conventional endovascular techniques, PED usage has similar efficacy and safety in treating small and medium-sized intracranial aneurysms (10). In a recent meta-analysis of stent-assisted coiling of aneurysms (11). the rate of complete occlusion at the final follow-up (9.03 ± 1.03 months) was reported to be between 73 and 88%, which is similar to our results. In addition, low retreatment rate is one of the reasons why PEDs are extensively used for small and medium-sized aneurysms. Malhotra et al. reported that PED treatment may have more health benefits due to its lower rates of retreatment compared to both simple coiling and stent-assisted techniques (12).

In our study, treatment of aneurysms with PEDs resulted in low major morbidity and mortality. Among the patients, early postoperative major ischemic stroke occurred in 1.8% (12/652), major hemorrhagic stroke in 1.2% (8/652), and neurogenic death in 0.8% (5/652). The combined major morbidity and mortality rate was 3.2% (21/652). In the PREMIER study (6), the combined major morbidity and mortality rate was 2.1% (3/140). Therefore, the major morbidity and mortality rates in our study were higher than that of the PREMIER study, which may have been caused by multiple factors. First, the interventional physician experience has an impact on complications. Jabbour et al. reported that the risk of complications decreased significantly as the physician experience increased (13). However, our data are from 2015, and the use of PED therapy for aneurysms is only in preliminary stages in China. Physician experience with PED use for aneurysm treatment is relatively limited, thus increasing the risk for complications. Second, of the aneurysms in our study, 12.3% (93/754) were posterior circulation aneurysms and 4.3% (32/754) were distal anterior circulation IAs. The study from Intre PED reported a higher rate of complications in posterior circulation aneurysms treated with PEDs (14). The complication rate in PED treatment for distal aneurysms is relatively high. A meta-analysis of 572 cases found a procedure-related morbidity of 9% and mortality of 4% (15).

In this study, we observed good device placement success rates. The device was successfully deployed in 99.7% (685/687) of stents, of which 3.8% (26/687) stents were successfully deployed after technical adjustment. In the PREMIER study, successful device deployment was reported in 99.3% (140/141) of cases. Additionally, in our study, 35.3% (266/754) of aneurysms were treated using PED-assisted coil embolization. The mean number of devices used per patient was 1.01 ± 0.12 (range: 1–2). Multiple devices were used in only 1.6% of cases. However, in the PREMIER study, five patients (3.5%) were treated with adjuvant coils, the average number of devices used per patient was 1.1 sie.3 (range: 0–2), and 6.4% of patients required multiple devices. In the Intre PED study, 28.0% of aneurysms ≤ 10 mm were treated using multiple stents (14). In terms of treatment strategies, pipeline-assisted coil embolization was more commonly chosen over multiple stents to treat aneurysms in this study. This choice of surgical strategy is determined by many factors. First, the use of multiple stents increases the financial burden of patients, so more coils are instead selected. Second, the use of multiple stents may affect the patency of branching vessels. Analysis of risk factors for complications suggested that the use of multiple stents increased the risk of complication (16). Third, coils adjunctive with PED are complementary, rather than competing, for cerebral aneurysm treatment (17). In a recent single-center study (18), with a very small subset of the same patients as the present study, it was reported that pipeline-assisted coiling increased the complete occlusion rate without increasing surgical complication rate. In terms of results, the surgical strategy we selected had similar results to the PREMIER study, which was more in line with China's national conditions.

There are some major limitations to this study worth noting. Specifically, this was a multi-center retrospective study, and there are biases in data collection as well as differences in surgical strategy decision among centers.

## Conclusion

This was a large sample, multicenter study that evaluated the safety and efficacy of PED use in the treatment of small and medium-sized intracranial aneurysms in China. In the largest study on PED treatment for small and medium-sized intracranial aneurysms to date, pipeline assisted coil embolization was chosen more often than multiple stents, with good results, high surgical success rates, high occlusion rates, and low morbidity and mortality.

## Data Availability Statement

The data analyzed in this study is subject to the following licenses/restrictions: To gain access, proposals should be addressed to the corresponding author. Requests to access these datasets should be directed to TL, dr.litianxiao@henu.edu.cn.

## Ethics Statement

The studies involving human participants were reviewed and approved by the Ethics Committee of Beijing Tiantan Hospital. Written informed consent to participate in this study was provided by the participants' legal guardian/next of kin.

## Author Contributions

PZ, XY, and TL: conception and design. HongqZ, JL, DS, YZ, SG, AM, YuW, WF, YaW, JW, GM, HS, and BL: acquisition of data. QS, KC, QZ, and YH: analysis and interpretation of data. HongyZ: drafting the article. LL: critically revising the article. All authors reviewed submitted version of manuscript.

## Funding

This study was sponsored by the Scientific and Technological Project of Henan Province (222102310208) and the Natural Science Foundation of Henan Province (222300420357).

## Conflict of Interest

The authors declare that the research was conducted in the absence of any commercial or financial relationships that could be construed as a potential conflict of interest.

## Publisher's Note

All claims expressed in this article are solely those of the authors and do not necessarily represent those of their affiliated organizations, or those of the publisher, the editors and the reviewers. Any product that may be evaluated in this article, or claim that may be made by its manufacturer, is not guaranteed or endorsed by the publisher.
