# Supplementary material for: Small and Medium-Sized Aneurysm Outcomes Following Intracranial Aneurysm Treatment Using the Pipeline Embolization Device: A Subgroup Analysis of the PLUS Registry
Source: Front Neurol. 2022 May 31;13:881353. doi: 10.3389/fneur.2022.881353 (PMC9197587; doi:10.3389/fneur.2022.881353)
Supplement: Supplementary file 1 [file Table_1.docx]

| Research trial | Study type | Study year | Number of aneurysms (subjects) | Mean aneurysm dome diameter | Mean angiographic follow-up time (% with DSA follow up) | Complete occlusion rate on follow-up angiography (time of follow-up) | Ischemic stroke | Neurologic morbidity and mortality |
| --- | --- | --- | --- | --- | --- | --- | --- | --- |
| PITA | Prospective multicenter trial | 2007-2008 | 3(31) | 11.5 | 6 mo (30/31) | 93.3% (at 6 mo) | 6.7% (2/30) | 6.7%Morbidity,  0% Mortality |
| PUFS | Prospective multicenter tria | 2008.11-2009.6 | 109(108) | 18.2 | 6 mo (97/107)  5 yr (61/107) | 86.8% (at 1 yr) 95.2% (at 5 yr) | 2.8% (3/107) | Morbidity4.7%(5/107)  Mortality2.8% (3/107) |
| Intre PED | Retrospective multicenter | 2008.7-2013.2 | 906(793) | 10.7 | Not reported | Not reported | 4.7% (37/793) | Morbidity7.4%(59/793)  Mortality3.8% (30/793)  Total 8.4% (67/793 |
| ASPIRe | Multicenter observational registry | Not reported | 207(191) | 14.5 | 7.8 mo 103/191 (54%) | 74.8% (at 7.8 mo) | 1.6% (3/191) | Morbidity6.8%(13/191) Mortality 1.6% (3/191)  Total 6.8% (13/191 |
| PREMIER | Prospective multicenter trial | 2015.7-2015.11 | 141(141) | 5.0 | 1 yr 138/141 (98%) | 81.9% (at 1 yr) | 0.7% (1) | Total 2.1% at 1 yr |
| PLUS | Retrospective multicenter | 2014.11-2019.10 | 1322(1171) | 12.79 | 6 (967/1322) | 81.4%(at 6 mo) | 3.8%（44/1171）early postoperative period,  0.6%(7)Postoperative follow-up period | 1.5%(17/1171) Mortality |

Supplementary table 1. Efficacy and Safety of Major Flow Diversion Trials for Intracranial Aneurysms
